# Effect of antioxidant supplementation on the reproduction of brown egg–layer breeders

**DOI:** 10.1016/j.psj.2026.107433

**Published:** 2026-07-16

**Authors:** Amanda de Moura Dani, Alexandre Pires Rosa, Cassiane Turchetto Ditadi, Julia Schneider, Daniele Cristina de Lima Escrobot, Guilherme Francisco Deda, Edita Sostaric Zandee

**Affiliations:** a Poultry Science Laboratory, Department of Animal Science, Federal University of Santa Maria, Avenida Roraima No. 1000, 97105–900, Santa Maria, Rio Grande do Sul, Brazil; b Impextraco Latin America, Ltda. Rua Curió, 312, 83707–760, Araucária, Paraná, Brazil; c Impextraco NV, Wiekevorstsesteenweg 38, 2220, Heist–op–den–Berg, Belgium

**Keywords:** Polyphenols, Reproduction, Brown egg–layer breeders, Sperm quality

## Abstract

Plant extracts rich in bioactive compounds, particularly polyphenols, have gained increasing attention in poultry breeder nutrition owing to their antioxidant and anti–inflammatory properties. These properties help preserve intestinal integrity and support improvements in gamete quality and embryonic viability. Commercial feed products, such as Elife®, have been developed to incorporate additives, including flavonoids, proanthocyanidins, and phenolic acids, delivering polyphenols in highly bioavailable forms and ensuring effective antioxidant activity. This study evaluated the effects of dietary supplementation with Elife® in brown egg–layer breeders (males and females) on productive and reproductive performance, egg quality, incubation traits, and progeny outcomes. Two experiments were conducted with birds aged from 54 to 70 weeks. In Experiment 1, 30 Rhode Island Red roosters were allocated to the following treatment groups: basal diet without additives (CON); basal diet + 0.5 kg of Elife®/t of feed (E500); and basal diet + 1 kg of Elife®/t of feed (E1000). Body weight, feed intake, and sperm quality were measured in each rooster every 28 days. For Experiment 2, 210 White Plymouth Rock brown egg–layer hens were assigned to the same three treatments as in Experiment 1. Body weight, feed intake, laying rate, egg quality, and incubation parameters were recorded every 28 days. Roosters receiving E1000 showed significantly improved sperm motility and an increased proportion of morphologically normal sperm without affecting feed intake, body weight, or sperm vigor. Hens under E1000 had lower feed intake than controls while maintaining egg quality (P > 0.05). Furthermore, combined parental supplementation with Elife® improved egg hatchability, reduced early embryonic mortality, and increased day–old chick weight. Overall, combined parental supplementation with Elife® demonstrated an effective nutritional strategy for improving fertility, embryonic viability, and chick quality in brown egg–layer breeders.

## Introduction

Plant–derived polyphenols have attracted significant attention in poultry farming owing to their recognized antioxidant, anti–inflammatory, and gut microbiota–modulating properties (Abd El–Hack et al., 2023; Li et al., 2023a, 2023b; Hafeez, 2024). In commercial layers, polyphenol supplementation has been associated with reduced lipid peroxidation in yolk and plasma, resulting in improved internal and external egg quality as well as enhanced production efficiency (Surai, 2014; Abd El–Hack et al., 2023; Herranz et al., 2024a; 2024b). Grape seed extract (from 500 to 750 mg/kg feed) has shown to improve laying rate, shell thickness, and shell weight while decreasing the levels of malondialdehyde, a key biomarker of oxidative stress (Khalil et al., 2022; Hafeez, 2024). Similarly, grape by–products have been found to increase yolk tocopherol content, thereby enhancing antioxidant stability and extending egg shelf life (Saleh et al., 2022; Herranz et al., 2024a; 2024b).

Other dietary antioxidants from diverse sources are crucial in neutralizing reactive oxygen species (**ROS**), thereby reducing oxidative damage to lipids, proteins, and DNA (Surai et al., 2019; Selim et al., 2021). The endogenous antioxidant defense system in poultry, primarily comprising superoxide dismutase (**SOD**), glutathione peroxidase (**GPx**), and catalase, can be potentiated by natural additives rich in bioactive compounds. These components promote gut health, immune function, fertility, and embryo quality (Surai, 2016; Abd El–Hack et al., 2020; Amer et al., 2023a; 2023b). Antioxidant supplementation becomes increasingly critical under adverse conditions, such as heat stress, high stocking density, or elevated metabolic demands, to preserve productive and reproductive performance and ensure superior hatchability and chick viability (Meng et al., 2020; Li et al., 2023a; 2023b).

Antioxidants have been widely studied in poultry nutrition, particularly vitamin E and selenium (Moreira, J., 2001; Silva, G. C., 2015; Batistioli, J. S., 2019), which synergize their effects to fortify cell membranes against lipid peroxidation and contribute directly to sperm viability and poultry fertility (Surai, 2016; Selim et al., 2021). Vitamin E is a potent fat–soluble antioxidant, crucial for sperm membrane integrity and maintenance of gamete quality, whereas selenium is a component of GPx, a key protective enzyme against oxidative stress (Surai et al., 2019). Furthermore, carotenoids, such as astaxanthin and lutein, preserve reproductive function, sperm motility, embryo viability, and hatchability (Amer et al., 2023a; 2023b). Another widely studied carotenoid that demonstrates antioxidant action and confers benefits in hatchability, fertility, and embryonic survival is canthaxanthin (Rosa et al., 2012a; 2012b; Bonilla et al., 2017; Rosa et al., 2017). The beneficial effects of maternal dietary antioxidants on progeny are well documented (Llaurado et al., 1997; Surai et al., 2003; Karadas et al., 2005; Surai, 2012; Rosa et al., 2017). For example, found that dietary supplementation with 6 ppm canthaxanthin in Ross breeders significantly reduced oxidative stress in their hatchlings. Furthermore, the synergistic benefits of synthetic and natural antioxidants are promising nutritional strategies for optimizing reproductive performance and chick quality in layer breeders.

Despite advancements in harnessing the benefits of natural antioxidants in poultry production, the effects of supplementation on brown egg–layer breeders have not been explored, particularly in male and female reproductive performance and the subsequent impacts on fertility, incubation, outcomes, and chick quality. Therefore, this study evaluated the productive and reproductive performance of brown egg–layer breeders under dietary supplementation with Elife®, a commercial supplement containing a standardized mixture of plant extracts, especially bioavailable polyphenols (including flavonoids, proanthocyanidins, and phenolic acids). Special emphasis was placed on egg quality, incubation traits, and sperm quality across different ages of birds.

## Materials and methods

### Ethics committee

Brown egg breeder hens were managed in accordance with the guidelines of the Ethics and Research Committee, under protocol number 6913131223, approved on February 6, 2024, at the Federal University of Santa Maria (UFSM), Santa Maria – RS, Brazil.

### Location and facilities

The study was conducted at the Poultry Science Laboratory of the Department of Animal Science at the Federal University of Santa Maria, Rio Grande do Sul, Brazil, in the second semester of 2024. Two experiments were performed to investigate the reproductive performance of brown egg–layer breeders. Experiment 1 evaluated roosters, whereas Experiment 2 evaluated hens.

### Treatments

The experimental treatments consisted of three dietary supplementation regimens of the antioxidant product Elife® (Impextraco NV, Heist–op–den–Berg, Belgium). The treatments were as follows: control, basal diet without Elife® supplementation (**CON**); basal diet supplemented with 0.5 kg of Elife® per ton of feed (**E500**); and basal diet supplemented with 1.0 kg of Elife® per ton of feed (**E1000**).

The same dietary supplementation regimens were applied to roosters (Experiment 1) and hens (Experiment 2) throughout the experimental period. The additive was thoroughly mixed into the basal diet to ensure homogeneous distribution before feeding to the birds. The supplementation levels were selected based on the manufacturer’s recommendations and the methodology of previous studies evaluating the use of plant–derived antioxidants in poultry diets.

### Experiment 1

Thirty Rhode Island Red breeder roosters were housed in cages measuring 0.33 × 0.60 × 0.60 m³ and equipped with automatic feeders and drinkers. The experimental phase spanned from 54 to 70 weeks of age and was divided into four 28–day periods. Water and feed were provided ad libitum. Body weight, feed intake, and sperm quality (including sperm motility, vigor, ejaculate volume, and sperm abnormality) were assessed every 28 days. The experimental design was completely randomized, with three treatments and 10 replicates per treatment, with one male per experimental unit.

### Nutrition and diets

The basal diet was formulated as a corn–soybean meal mash. The diets contained no specific antioxidant additives or mycotoxin binders. A commercial premix with reduced levels of vitamin E, vitamin C, and selenium was used to minimize the potential antioxidant contributions of these nutrients. The inclusion levels of these vitamins and minerals were maintained at the minimum levels recommended by the National Research Council NRC (1994) for breeder roosters. This procedure was applied to the diets in both experiments. Diet formulation and nutritional levels followed the standard recommendations routinely used for males at Poultry Laboratory of the Federal University of Santa Maria (Table 1). The basal diets were supplemented with varying doses of Elife®.

**Table 1:** Basal diet composition and nutritional levels for males and females.

	Male diet	Female diet
**Ingredients**		
Corn 8.10%	61.52	62.54
Soybean meal, 46%	14.59	22.11
Limestone	1.75	9.73
Wheat bran	15.00	2.82
Dicalcium phosphate	1.56	1.09
Soybean oil	––––	0.50
Salt	0.400	0.35
DL–methionine, 99%	0.078	0.24
Vitamin and mineral premix^1^	0.600	0.60
Choline chloride 60%	0.060	0.06
Vitamin E 50%	0.004	0.004
Inert material	4.67	–––
Total (kg)	100.00	100.00
**Calculated nutrient composition**		
AMEn (kcal/kg)	2,700	2,750
Crude protein (%)	14.00	16.00
Dig. LYS (%)	0.56	0.70
Dig. MET + CYS (%)	0.49	0.70
Dig. MET (%)	0.28	0.47
Dig. THR (%)	0.45	0.53
Dig. TRP (%)	0.14	0.16
Dig. VAL (%)	0.56	0.65
Ca (%)	1.00	4.00
Available P (%)	0.45	0.33
Na (%)	0.19	0.17
Linolenic acid (%)	1.50	1.66

1 Provided the following per kilogram of premix: vitamin A. 1,610,000 IU/kg; vitamin E 4,000 IU/kg; vitamin D3 600,000 IU/kg; vitamin K3 408 mg/kg; vitamin B1 303 mg/kg; vitamin B12 2,400 mcg/kg; vitamin B2 888 mg/kg; vitamin B6 613 mg/kg; folic acid 120 mg/kg; biotin 16 mg/kg; pantothenic acid 2,001 mg/kg; copper 1,200 mg/kg; iron 10 g/kg; iodine 155 mg/kg; manganese 12.1 g/kg; selenium 50 mg/kg; and zinc 10 g/kg.

### Analyzed parameters

The animals were evaluated daily by recording their body weight and feed intake. The feed conversion ratio was calculated for each 28–day period. Semen quality was analyzed immediately after semen collection, along with sperm motility, vigor, and morphology.

Semen was collected in pre–labeled, sterile 20–mL plastic Falcon tubes with conical bottoms. Sperm morphology evaluation, semen samples were diluted at a ratio of 1:500 in a buffered saline solution. An aliquot of the diluted sample was placed on a clean glass slide, smeared, and air–dried. The slides were subsequently stained using a standard eosin–nigrosin technique to allow proper visualization of sperm cells. A total of 200 sperm cells per sample were examined under a light microscope. Cells were classified as normal or abnormal according to morphological criteria described by, considering abnormalities in the head, midpiece, and tail regions. The percentage of abnormal cells was then calculated.

Motility was defined as the percentage of motile spermatozoa and was objectively determined using a computer–assisted sperm analysis (CASA) system (AndroScope, Version 1.1.1.6; Minitube, Tiefenbach, Germany). Semen samples were diluted prior to analysis, maintained at 32°C, loaded into specific counting chambers immediately after collection, and analyzed according to the manufacturer's recommendations. Sperm vigor was assessed independently by two trained evaluators using a light microscope and scored on a subjective scale from 0 to 5 according to the predominance and intensity of progressive forward movement. Both evaluators were blinded to the dietary treatments to minimize observer bias, and the final vigor score corresponded to the mean of the two independent evaluations.

### Experiment 2

In total, 210 White Plymouth Rock brown egg–layer breeders from the LAVIC–UFSM were housed in cages measuring 0.33 × 0.45 × 0.40 m³, which were equipped with feeders, drinkers, and an egg collection system. The experimental phase spanned from 54 to 70 weeks of age and was divided into four 28–day periods. As in Experiment 1, water and feed were provided ad libitum.

The experiment was arranged in a completely randomized design with three treatments and 10 replicates, with 7 females per experimental unit.

***Nutrition and Diets.*** The basal diet was formulated as a corn–soybean meal mash and was free of antioxidants or mycotoxin adsorbents. Dietary formulation and nutritional levels followed the standard protocols for females implemented at LAVIC–UFSM (Table 1). Treatments consisted of varying doses of Elife®.

***Analyzed Parameters.*** The analyzed parameters included performance, egg quality, and incubation traits. Performance was analyzed by measuring feed intake and body weight at 4–week intervals. Eggs were collected four times daily. The health status of the experimental flock was recorded daily based on clinical observations, including mortality, morbidity, behavioral changes, and the presence of clinical signs such as respiratory distress, diarrhea, and locomotor abnormalities.

Egg quality was evaluated at 58, 62, 66, and 70 weeks of age. During the last 3 days of each period, 3 eggs per replicate were collected. Egg, albumen, yolk, and shell weights were measured using a precision scale (0.001 g). Haugh Unit, vitelline membrane strength, and eggshell strength were assessed using a texture analyzer. Yolk color was analyzed using the DSM Yolk Color Fan. Albumen and yolk pH were measured using an AKSO AK90 digital pH meter. Specific gravity was determined by immersing the eggs in saline solutions with densities ranging from 1.065 to 1.095 g/cm³ (in increments of 0.005) (Hamilton, 1982).

Hen fertility and egg incubation parameters were evaluated over 16 consecutive incubation cycles. Hens were artificially inseminated once a week with semen collected from roosters belonging to the same dietary treatment group. Because hens were inseminated exclusively with semen obtained from roosters receiving the corresponding dietary treatment, the incubation responses (fertility, hatchability and embryonic mortality) represent the combined effect of dietary supplementation in both male and female breeders and should therefore be interpreted as parental treatment effects rather than exclusively maternal effects.

Eggs were collected four times daily to avoid prolonged exposure and potential damage. Eggs from each experimental replicate (one cage containing seven hens) were individually identified according to replicate and dietary treatment at collection. During each of the 16 consecutive weekly incubation cycles, eggs from all experimental replicates and treatments were incubated simultaneously in a multi–stage incubator under identical environmental conditions. At 18 days of incubation, eggs were transferred to the hatcher and placed in separate trays according to their respective experimental replicate. Fertility, hatchability and embryonic mortality were subsequently calculated for each experimental replicate, which remained the experimental unit throughout the incubation process and statistical analyses. Hatching eggs were stored for a maximum of 7 days in a temperature–controlled room (from 18°C to 20°C; from 75% to 80% relative humidity) until incubation.

Eggs were incubated in multi–stage incubators (Casp, Amparo, SP, Brazil) at 37.5°C and 60% relative humidity. On day 18 of incubation, the eggs were transferred to a hatcher maintained at approximately 36.5°C and 65% relative humidity. On day 21, chicks were removed from the hatcher, counted, and individually weighed.

The eggs were incubated weekly and evaluated after 21 days. Any unhatched eggs were analyzed for residues and evaluated as described previously (Rosa et al., 2012a; 2012b). The eggs were evaluated via visual macroscopic examination for infertility; embryonic mortality up to the first 48 h of incubation (EM1); embryonic mortality occurring between 3 and 7 days (EM2); embryonic mortality occurring between 8 and 14 days of incubation (EM3); mortality occurring between 15 and 21 days of incubation, including dead pipped eggs (EM4); and live pipped eggs (eggs with broken shells where the embryo was unable to exit but was still alive upon removal from the hatcher). Eggs with internal changes in color and odor were considered contaminated. Hatchability (total hatched eggs/total incubated eggs), hatchling fertile eggs, and fertility rates were also analyzed. Chick weight was evaluated upon hatching. Chicks were individually weighed using a precision scale.

### Flock management

Mortality was monitored daily in the entire flock and recorded. Dead birds were removed from the cages, weighed individually on a digital scale, and properly disposed of in accordance with institutional sanitation protocols.

Environmental conditions in the experimental house were monitored daily by recording the minimum and maximum temperatures. Curtain management was adjusted based on ambient temperature and bird behavior, with curtains closed during colder periods to maintain adequate thermal comfort.

Standard management practices, including routine cleaning, disinfection, and vaccination protocols recommended for breeder flocks, were implemented throughout the experiment. Each experimental unit was equipped with a dedicated, labeled feed container. Feed was manually supplied daily, and feeders were emptied and weighed every 4 weeks to determine feed intake for each experimental phase.

### Statistical analysis

Data were analyzed using the General Linear Model (GLM) procedure of SAS software (SAS Institute Inc., Cary, NC, USA). The experimental period was prospectively divided into four predefined 28–day production phases. Statistical analyses were performed independently for each production phase because each phase represented a biologically distinct stage of breeder performance, allowing treatment comparisons within each production phase.

For each production phase, the experimental unit consisted of one rooster in Experiment 1 and one cage containing seven hens in Experiment 2, with one observation per experimental unit included in each statistical analysis.

For the overall experimental period (54 to 70 weeks of age), the arithmetic mean of the four evaluation periods was calculated for each experimental unit and subsequently used as the response variable in the cumulative analysis. Therefore, the cumulative analysis was also based on a single observation per experimental unit. Treatment means were compared using Tukey's multiple comparison test, and differences were considered statistically significant at P ≤ 0.05.

## Results and discussion

### Experiment 1

Table 2 presents the results of Experiment 1. Dietary supplementation with Elife® did not affect rooster body weight or feed intake at any phase during the experimental period (P > 0.05).

**Table 2:** Performance and sperm quality of roosters with or without Elife® supplementation.

**Parameters**	Dietary treatments			Average	SEM	CV (%)	P–value
	kg Elife®/T feed						
	None	0.5	1.0				
	CON	E500	E1000				
**Period 1** (from 54 to 58 weeks)							
Body weight (g)	2,957	2,906	2,893	2,918	164.62	5.64	0.6613
Feed intake (g/bird/day)	98.72	96.69	93.19	96.20	9.73	12.24	0.3267
Sperm motility (%)	84.3 b	93.0 ab	96.2 a	91.16	7.92	8.68	0.0068
Sperm vigor	3.7	3.7	3.5	3.6	0.91	25.23	0.8541
Normal spermatozoa (%)	85.38 b	89.39 b	95.20 a	89.99	3.80	4.22	<0.0001
**Period 2** (from 58 to 62 weeks)							
Body weight (g)	3,045	2,945	2,937	2,975	198.44	6.66	0.4074
Feed intake (g/bird/day)	97.37	94.54	98.22	96.71	11.43	13.19	0.7548
Sperm motility (%)	80.5 b	90.7 a	96.0 a	89.06	6.10	6.85	<0.0001
Sperm vigor	4.4	3.8	3.8	4.0	0.97	24.34	0.2983
Normal spermatozoa (%)	87.75 b	91.60 b	97.30 a	92.21	4.37	4.74	0.0002
**Period 3** (from 62 to 66 weeks)							
Body weight (g)	2,776	2,753	2,758	2,762	247.10	8.94	0.4074
Feed intake (g/bird/day)	92.95	97.15	90.80	93.63	13.25	19.79	0.4271
Sperm motility (%)	84.6 b	90.2 ab	96.6 a	90.46	9.27	10.25	0.0260
Sperm vigor	3.9	3.5	3.7	3.7	1.14	30.99	0.7402
Normal spermatozoa (%)	88.70 b	90.35 a	96.05 a	91.70	4.47	4.87	0.0027
**Period 4** (from 66 to 70 weeks)							
Body weight (g)	2,795	2,767	2,775	2,779	241.89	8.70	0.4994
Feed intake (g/bird/day)	91.34	92.04	97.71	93.69	18.76	20.76	0.1907
Sperm motility (%)	85.3 b	90.3 ab	96.4 a	90.66	7.67	8.46	0.0118
Sperm vigor	3.9	4.2	4.1	4.0	0.64	15.97	0.5818
Normal spermatozoa (%)	87.50 b	91.35 ab	96.30 a	91.71	4.84	5.27	0.0016
**Total experimental period** (from 54 to 70 weeks)							
Total body weight (g)	2,893	2,843	2,840	2,859	175.14	6.12	0.7508
Feed intake (g/bird/day)	95.09	95.11	94.98	95.06	6.37	6.70	0.9987
Sperm motility (%)	83.67 c	91.05 b	96.30 a	90.34	4.34	4.80	<0.0001
Sperm vigor	3.97	3.80	3.77	3.85	0.58	15.27	0.7124
Normal spermatozoa (%)	87.50 b	91.35 ab	96.30 a	91.40	2.61	2.85	<0.0001

Tukey’s test a > b > c.

During Period 1 (from 54 to 58 weeks), sperm motility (P = 0.0068) and the proportion of normal sperm morphology (P < 0.001) significantly improved in roosters receiving Elife® supplementation. In particular, sperm motility values were higher in the E500 group than in the control group. Thus, the antioxidant compounds present in Elife® may exert a protective effect on sperm cells by helping to mitigate oxidative damage associated with reproductive aging. The observed improvement in sperm motility may reflect better preservation of mitochondrial integrity and membrane fluidity, both of which are essential for progressive motility. Polyphenols, the primary bioactive components of Elife®, have been reported to scavenge reactive oxygen species (ROS) and modulate the activity of endogenous antioxidant enzymes, such as superoxide dismutase (SOD) and glutathione peroxidase (GPx) (Surai et al., 2019). Therefore, one possible explanation for the improved sperm quality observed in the present study is that antioxidant supplementation may have contributed to reducing oxidative damage and preserving plasma and mitochondrial membrane functionality, thereby maintaining sperm motility and viability. However, oxidative stress biomarkers and antioxidant enzyme activities were not evaluated in the present study, and these mechanisms should therefore be regarded as plausible hypotheses based on previous literature rather than direct evidence.

Furthermore, the higher proportion of morphologically normal spermatozoa may also be associated with improved spermatogenesis and spermiogenesis, processes known to be highly sensitive to oxidative stress. Partyka et al., 2021 reported that prolonged exposure of germ cells to ROS induces tail and acrosome abnormalities, compromising fertilizing potential. Therefore, although the present findings are consistent with the proposed antioxidant effects of dietary polyphenols, the mechanisms underlying these improvements remain speculative and require confirmation through direct evaluation of oxidative biomarkers.

During Period 2 (from 58 to 62 weeks), sperm motility remained significantly higher in roosters fed diets supplemented with E500 and E1000 than in the CON group (P < 0.0001). Similarly, the proportion of morphologically normal spermatozoa was higher in the E500 and E1000 groups than in the control group (P = 0.002).

Preserved motility and morphological integrity throughout a 16–week period support the hypothesis that the polyphenols in Elife® protect cell organelles against progressive oxidative damage. This finding is particularly true for mitochondria and plasma membranes, structures that are highly vulnerable to lipid peroxidation due to their high content of polyunsaturated fatty acids (Surai et al., 2019; Partyka et al., 2021). According to Rosa et al., 2012 and Bonilla et al., 2017, prolonged antioxidant supplementation is essential to delay fertility decline in aging roosters, as increased ROS production in the reproductive tract compromises both spermatogenesis and sperm motility.

These results suggest that the sustained activity of the phenolic compounds present in Elife® helps maintain redox balance, thereby preserving mitochondrial function and sperm structural integrity. This oxidative stability may also enhance sperm maturation processes within the avian vas deferens and epididymis, resulting in a more functional and homogeneous sperm population. Consequently, continuous Elife® supplementation may help preserve the fertilizing potential of breeders, even during the natural age–related physiological decline.

In Period 3 (from 62 to 66 weeks), roosters supplemented with E1000 maintained significantly higher sperm motility than the control group (P = 0.0260), exceeding 96% total motility. Similarly, the percentage of morphologically normal sperm was higher in birds receiving E500 and E1000 (P = 0.0027). These results are particularly significant because reproductive aging in roosters is typically associated with a progressive decline in sperm motility and an increase in morphological abnormalities (Lake, 1989; Ansari et al., 2020). Therefore, the preservation of high motility and normal morphology at this advanced age suggests that Elife® supplementation mitigated the harmful effects of oxidative stress commonly associated with reproductive senescence.

This improvement indicates that continuous supplementation with polyphenolic compounds exerts a cumulative protective effect on the reproductive system by attenuating testicular and epididymal oxidative stress, thereby preserving spermatogenesis and sperm function (Rosa et al., 2012a; 2012b; Surai et al., 2019). One possible explanation for the improvements observed in sperm quality is the antioxidant activity of polyphenols, which has been associated with the stabilization of cellular and mitochondrial membranes and reduced lipid peroxidation in previous studies (Surai et al., 2019). However, these mechanisms remain hypothetical in the context of the present study, as oxidative biomarkers and sperm DNA integrity were not evaluated.

Moreover, the positive response observed even in roosters over 60 weeks of age suggests that continuous dietary antioxidant supplementation may help mitigate the harmful effects of reproductive aging, thereby extending the reproductive longevity of breeders. According to Bonilla et al., 2017, the balance between ROS and endogenous antioxidants is critical for fertility in older birds, which can be partially restored by supplementation with phenolic compounds.

The favorable results observed in previous periods persisted in Period 4 (from 66 to 70 weeks). Sperm motility (P = 0.0118) and the percentage of morphologically normal sperm (P = 0.0016) remained significantly higher in roosters supplemented with E500 and E1000 than in the control group. Although no significant differences in sperm vigor were observed among treatments in any of the evaluated periods (P > 0.05), the consistent improvement in sperm motility and morphology observed throughout the experimental period suggests that dietary supplementation with Elife® helps mitigate the physiological decline in reproductive function associated with advancing age.

Phenolic compounds and other dietary antioxidants are fundamental to maintaining cell membrane integrity and mitochondrial function in spermatozoa by scavenging ROS and preventing lipid peroxidation (Surai et al., 2019). Accordingly, the addition of Elife® to the diet may have exerted a cumulative and sustained beneficial effect, possibly contributing to the maintenance of redox balance and the preservation of sperm viability and functionality, even in aging roosters. Surai and Fisinin (2014) found that reduced endogenous antioxidant capacity contributes significantly to fertility decline in aging breeders, underscoring the strategic role of dietary supplementation with exogenous antioxidants to maintain semen quality and extend reproductive lifespan.

Throughout the experimental period (from 54 to 70 weeks), dietary supplementation with Elife® significantly improved sperm motility, with birds receiving E500 and E1000 exhibiting superior results compared with the control group (P < 0.0001). The percentage of morphologically normal sperm was also higher in roosters supplemented with E1000 than in the control group (P < 0.0001), whereas body weight, feed intake, and sperm vigor were not affected by the treatments (P > 0.05). These results confirm the potential of polyphenols to preserve sperm quality over time, thereby prolonging the reproductive performance of aging roosters. Thus, supplementation with natural antioxidants represents a promising nutritional strategy to mitigate the effects of oxidative stress on spermatogenesis, sperm DNA integrity, and fertility in aging males (Rosa et al., 2012a, 2012b; Surai et al., 2019).

Collectively, these results indicate that continuous supplementation with natural antioxidants exerts a cumulative protective effect on semen quality, sustaining reproductive performance even in older birds.

### Experiment 2

The results of Experiment 2 (Table 3) indicate that dietary antioxidant supplementation significantly augmented feed intake (P < 0.05) across all phases of the experimental period (from 54 to 70 weeks of age). Birds in the E500 group exhibited lower feed intake than those in the CON group across all four periods (Period 1, P = 0.0006; Period 2, P < 0.0001; Period 3, P < 0.0001; Period 4, P < 0.0001) and the total period (P < 0.0001). Varying antioxidant concentrations influenced bird feed intake across most evaluation periods.

**Table 3:** Reproductive performance, egg quality, incubation parameters, and progeny of breeders with or without Elife® supplementation.

**Parameters**	Dietary Treatments			Average	SEM	CV (%)	P–value
	kg Elife®/T feed						
	None	0.5	1.0				
	CON	E500	E1000				
**Period 1 (from 54 to 58 weeks)**							
Body weight (g)	2,006	2,008	2,006	2,007	267.23	13.59	0.4790
Feed intake (g/bird/day)	103.48 a	99.43 b	99.94 ab	100.95	7.14	7.08	0.0006
Egg laying rate (%)	63.61	62.50	68.92	65.01	6.38	9.85	0.0728
Egg weight (g)	59.17	53.31	58.67	57.05	4.75	8.33	0.1083
Albumen weight (g)	34.49	35.66	38.32	36.16	6.80	18.82	0.4466
Yolk weight (g)	19.27	16.01	18.41	17.90	2.91	16.25	0.0593
Egg shell weight (g)	5.40	4.86	5.13	5.12	0.61	12.08	0.1496
Haugh Unit	69.74	70.78	67.89	69.47	12.86	11.26	0.8793
Yolk color	5.2	5.1	5.1	5.13	1.07	21.00	0.9718
Vitelline membrane (N)	5.05	5.57	5.43	5.35	0.90	16.82	0.4211
Shell breaking force (N)	32.86	32.65	34.03	33.18	11.21	33.79	0.9571
Yolk pH	6.32	6.01	6.39	6.24	0.49	7.96	0.2100
Albumen pH	8.43	8.50	8.42	8.45	0.29	3.45	0.8013
Specific gravity (g/cm³)	1,076	1,072	1,074	1,074	4.47	0.41	0.1549
Hatchability rate (%)	52.00 b	56.48 ab	64.68 a	57.72	8.69	15.05	0.0101
Pipped eggs (%)	1.58	1.79	1.70	1.69	1.21	71.94	0.9264
Mortality 1 (%)	36.44 a	29.28 ab	22.76 b	29.49	8.04	27.28	0.0031
Mortality 2 (%)	2.36	4.14	2.96	3.15	1.59	50.52	0.0565
Mortality 3 (%)	1.25	2.12	2.47	1.94	1.43	73.76	0.1693
Mortality 4 (%)	3.14	2.75	3.17	3.02	2.25	74.44	0.8946
Infertility (%)	3.19	3.42	2.65	3.09	2.66	86.17	0.8039
Chick body weight (g)	35.99 b	37.63 ab	38.41 a	37.34	1.89	4.99	0.0226
**Period 2 (from 58 to 62 weeks)**							
Body weight (g)	2,097	2,085	2,034	2,072	289.50	12.46	0.2508
Feed intake (g/bird/day)	105.36 a	99.32 b	104.67 ab	103.12	8.99	8.72	<0.0001
Egg laying rate (%)	60.00	56.74	63.21	59.98	7.37	12.29	0.1653
Egg weight (g)	58.36	60.13	60.56	59.68	5.92	9.92	0.6821
Albumen weight (g)	35.74	36.90	36.64	36.42	4.23	11.62	0.8130
Yolk weight (g)	17.24	17.80	18.26	17.76	2.06	11.61	0.5487
Egg shell weight (g)	5.38	5.42	5.65	5.48	0.64	11.80	0.6015
Haugh Unit	94.59	93.10	94.17	93.96	8.00	18.51	0.9125
Yolk color	5.5	5.4	5.5	5.46	0.84	15.50	0.9547
Vitelline membrane (N)	5.44	5.84	5.90	5.72	1.33	23.25	0.7061
Shell breaking force (N)	37.77	39.13	39.77	38.89	7.10	18.26	0.8132
Yolk pH	6.39	6.37	6.33	6.36	0.23	3.69	0.8454
Albumen pH	8.20	8.20	8.21	8.20	0.20	3.49	0.9921
Specific gravity (g/cm³)	1,081	1,081	1,081	1,081	5.55	0.51	0.9734
Hatchability rate (%)	46.85	41.79	48.70	45.78	10.32	22.54	0.3160
Pipped eggs (%)	2.28	2.90	2.11	2.43	1.78	73.26	0.5899
Mortality 1 (%)	38.31	40.71	33.85	37.62	10.05	26.71	0.3172
Mortality 2 (%)	3.90	4.52	5.35	4.59	2.46	53.75	0.4338
Mortality 3 (%)	2.33	3.24	2.19	2.59	2.18	84.46	0.5108
Mortality 4 (%)	2.75	2.71	3.54	3.00	2.41	80.29	0.6895
Infertility (%)	3.53	4.09	4.22	3.95	2.69	68.18	0.8331
Chick body weight (g)	36.79 b	39.25 a	38.42 ab	38.15	1.72	4.50	0.0117
**Period 3 (from 62 to 66 weeks)**							
Body weight (g)	2,029	2,003	2,004	2,012	247.80	12.38	0.3422
Feed intake (g/bird/day)	100.06 a	91.35 c	96.91 b	96.11	22.32	27.52	<0.0001
Egg laying rate (%)	52.09	49.59	57.85	53.18	8.45	15.88	0.0999
Egg weight (g)	60.68	58.55	59.54	59.59	4.61	7.74	0.5920
Albumen weight (g)	36.46	34.19	35.89	35.51	3.98	11.22	0.4277
Yolk weight (g)	19.33	19.50	18.63	19.16	2.20	11.51	0.6508
Egg shell weight (g)	4.89	4.85	5.00	4.91	0.35	7.29	0.6094
Haugh unit	81.37	80.47	79.11	80.32	11.09	8.51	0.9002
Yolk color	5.1	5.3	5.1	5.16	1.01	19.67	0.8795
Vitelline membrane (N)	6.07	5.46	6.57	6.02	1.69	28.16	0.3247
Shell breaking force (N)	45.57	49.40	47.09	47.35	9.96	21.03	0.6906
Yolk pH	6.23	6.16	6.02	6.13	0.22	3.65	0.1229
Albumen pH	7.97	7.97	7.98	7.97	0.27	3.41	0.9955
Specific gravity (g/cm³)	1,077	1,073	1,076	1,075	4.14	0.38	0.1444
Hatchability rate (%)	37.23	38.44	44.54	40.70	11.52	28.76	0.3301
Pipped eggs (%)	2.75	5.34	3.70	3.93	3.27	83.25	0.2189
Mortality 1 (%)	35.34	31.46	29.92	32.24	10.29	31.91	0.4877
Mortality 2 (%)	5.81	4.18	5.61	5.20	3.57	68.67	0.5488
Mortality 3 (%)	7.40	7.98	6.28	7.22	4.59	63.60	0.7060
Mortality 4 (%)	2.75	2.71	3.54	3.00	3.48	42.76	0.1572
Infertility (%)	2.60	3.31	3.54	3.15	2.78	88.19	0.7355
Chick body weight (g)	36.18 b	37.47 ab	38.24 a	37.30	1.30	3.48	0.0052
**Period 4 – (from 66 to 70 weeks)**							
Body weight (g)	2,091	2,064	2,032	2,062	245.86	11.92	0.3134
Feed intake (g/bird/day)	101.55 a	93.21 b	96.93 b	97.23	10.48	10.78	<0.0001
Egg laying rate (%)	48.21	44.46	52.67	48.45	10.74	22.16	0.2487
Egg weight (g)	58.01	57.65	57.71	57.79	5.02	8.70	0.9853
Albumen weight (g)	35.66	35.08	35.31	35.35	3.90	11.04	0.9459
Yolk weight (g)	17.39	17.35	17.15	17.30	2.08	12.04	0.9610
Egg shell weight (g)	4.95	5.19	5.24	5.13	0.46	9.05	0.3366
Haugh Unit	68.19	66.59	73.97	69.58	10.93	13.80	0.2994
Yolk color	5.2	5.0	5.0	5.06	0.59	11.76	0.6908
Vitelline membrane (N)	5.14	4.33	4.98	4.81	1.45	30.27	0.4321
Shell breaking force (N)	43.09	36.97	39.55	39.87	10.05	25.22	0.4057
Yolk pH	6.23	6.27	6.26	6.25	0.19	3.05	0.8886
Albumen pH	8.10	8.23	8.16	8.16	0.24	3.02	0.5079
Specific gravity (g/cm³)	1,076	1,079	1,078	1,078	5.53	0.51	0.4768
Hatchability rate (%)	37.06	42.31	44.54	41.03	10.69	25.88	0.2913
Pipped eggs (%)	2.84	2.90	3.28	3.01	2.64	87.93	0.9225
Mortality 1 (%)	44.95 a	36.49 ab	35.06 b	38.83	9.33	24.02	0.0451
Mortality 2 (%)	3.43	3.53	3.28	3.41	2.08	61.06	0.9636
Mortality 3 (%)	3.20	4.84	3.03	3.69	2.50	67.75	0.2218
Mortality 4 (%)	2.37	3.40	4.19	3.32	2.93	88.29	0.3936
Infertility (%)	6.12	6.51	6.59	6.40	4.54	70.83	0.9704
Chick body weight (g)	36.14 b	37.07 ab	38.15 a	37.12	1.17	3.16	0.0028
**Total experimental period (from 54 to 70 weeks)**							
Total body weight (g)	2,055	2,039	2,018	2,038	236.24	11.59	0.6115
Feed intake (g/bird/day)	102.61a	95.82c	99.61b	99.35	5.56	5.56	<0.0001
Egg laying rate (%)	55.98	53.32	60.66	56.65	7.39	13.04	0.0983
Hatchability rate (%)	43.28	44.76	50.62	46.22	7.69	16.65	0.0977
Pipped eggs (%)	2.36	3.23	2.70	2.76	1.26	45.77	0.3166
Mortality 1 (%)	38.76 a	34.49 ab	30.40 b	34.55	6.55	18.97	0.0286
Mortality 2 (%)	3.87	4.09	4.30	4.09	1.21	29.75	0.7406
Mortality 3 (%)	3.54	4.54	3.49	3.86	1.68	43.68	0.3061
Mortality 4 (%)	4.28	4.52	4.32	4.37	1.52	34.73	0.9281
Infertility (%)	3.86	4.33	4.25	4.15	1.93	46.57	0.8462
Chick body weight (g)	36.28 b	37.86 a	38.31 a	37.48	0.96	2.57	0.0002

Tukey’s test a > b > c.

Throughout the study period, the supplementation diets did not significantly affect (P > 0.05) laying rate, egg weight, yolk, albumen, or shell weights, Haugh Unit, yolk color, vitelline membrane or eggshell breaking strength, albumen pH, or specific gravity.

In Period 1 (from 54 to 58 weeks), a significant increase (P = 0.0101) in hatchability was observed in birds supplemented with E1000 (64.68%) compared with those in CON (52.00%). This trend persisted across the other periods but was not significant (P > 0.05). However, during the entire experimental period (from 54 to 70 weeks of age), this effect was distinct: CON birds had a hatchability of 43.28%, whereas E1000 birds reached 50.62% (P = 0.0477).

Chick hatching weight was also significantly influenced by maternal dietary antioxidant supplementation. Across all evaluation periods and throughout the entire experimental period (from 54 to 70 weeks of age), antioxidant supplementation significantly altered hatch weight (P < 0.05). Birds fed E1000 produced heavier chicks than CON birds (Period 1, P = 0.0226; Period 2, P = 0.0117; Period 3, P = 0.0052; Period 4, P = 0.0028; and the total period, P = 0.0002). This finding further confirms the consistent positive effects of supplementation over time.

Furthermore, the higher hatching weight of chicks in E1000 than those in CON highlights the positive effect of supplementation on embryo quality and nutrient transfer efficiency from the egg to the embryo. Higher hatching weights are associated with greater vitality and superior early growth performance (Wilson, 1991; Surai et al., 2019). These effects may be attributed to enhanced deposition of antioxidant nutrients and minerals in the egg, thereby increasing the availability of essential compounds for embryonic metabolism (Surai et al., 2021).

These results likely reflect the combined effects of parental antioxidant supplementation. Maternal antioxidant deposition into the egg may have contributed to improved embryonic development, while the improvement in semen quality observed in Experiment 1 may also have enhanced fertilization success and early embryonic viability. Increased hatching weight may reflect better embryonic development, a consequence of a more stable intra–egg environment with less oxidative stress during incubation (Zhandi et al., 2020; Asa, 2022). Surai et al., 2019 found that providing dietary antioxidants to breeders can increase the deposition of vitamin E, carotenoids, and phenolic compounds in the yolk, thereby ensuring mitochondrial protection and embryonic integrity. Higher glycogen reserves and yolk absorption yield heavier chicks, and both are associated with lower mortality in the first week of life (Yadgary and Uni, 2012).

Furthermore, Yang et al. (2020) observed that antioxidant supplementation in breeder hens increased chick vigor, uniformity, and viability, findings consistent with those of the present study. Accordingly, the E1000 group exhibited lower early embryonic mortality (0–48 h of incubation) than the CON group. These findings are consistent with the hypothesis that parental antioxidant supplementation may contribute to a more favorable environment for early embryonic development, as suggested in previous studies. However, the mechanisms underlying this response were not directly evaluated in the present study and therefore remain speculative.

These outcomes may be associated with the combined parental supplementation strategy. Maternal deposition of antioxidant compounds into the egg probably contributed to embryo protection, whereas the improved semen quality observed in supplemented roosters may also have enhanced fertilization capacity and early embryonic development (Surai, 2016). The observed increase in chick weight during this phase, characterized by a marked natural decline in breeder reproductive performance, indicates that combined parental supplementation with Elife® exerts a protective effect on reproductive metabolism and embryonic viability.

In the first (from 54 to 58 weeks of age), fourth (from 66 to 70 weeks), and total experimental (from 54 to 70 weeks) periods, embryos from hens given E1000 presented lower EM1 than those from CON breeders. In Period 1, the E1000 group had an EM1 of 22.76%, whereas the CON group had an EM1 of 36.44% (P = 0.0031). In Period 4, EM1 was 44.95% for CON but 35.06% for E1000 (P = 0.0451). This decrease in early EM1 is evident throughout the study period. A considerable reduction was observed in early embryonic mortality (EM1) in embryos from E1000 breeders (30.40%) compared with those from CON breeders (38.76%). The lower mortality during the initial stages of incubation is biologically relevant, as early embryonic development is highly susceptible to oxidative stress because of the high metabolic rate and rapid free formation during cell differentiation (Surai et al., 2019).

The lower embryonic mortality observed with E1000 supplementation may be associated with a more favorable environment for embryonic development, as suggested in previous studies evaluating dietary antioxidants in poultry. One possible explanation is that antioxidant supplementation may contribute to maintaining redox balance through the modulation of endogenous antioxidant defenses, including enzymes such as glutathione peroxidase (GPx) and superoxide dismutase (SOD), which have been associated with the maintenance of embryonic membrane integrity and tissue viability (Araújo and Lara, 2023).

Overall, the present findings are consistent with previous studies reporting beneficial effects of dietary antioxidants on cellular protection against reactive oxygen species (ROS), as well as improvements in fertility, hatchability, and chick quality (Rosa et al., 2012a; 2012b; Surai, 2016; Bonilla et al., 2017; Surai et al., 2019). Therefore, parental supplementation with Elife® may represent a promising nutritional strategy to support reproductive performance, particularly during the later stages of the production cycle, when reproductive aging naturally compromises fertility and hatchability.

## Conclusions

Dietary supplementation with Elife® (1 kg/t) in brown egg–layer breeders yielded consistent and measurable improvements in reproductive performance. In females, Elife® reduced feed intake without altering egg quality, whereas supplementation increased sperm motility and the proportion of morphologically normal sperm in males, enhancing semen quality. Breeders receiving Elife® supplementation also produced chicks with lower early embryonic mortality and higher hatch weights. Collectively, these findings demonstrate that supplementation with 1 kg/t Elife® enhances parental reproductive parameters and fortifies the early development of progeny in brown egg–layer breeders.

## CRediT authorship contribution statement

**Amanda de Moura Dani:** Writing – review & editing, Writing – original draft, Software, Methodology, Investigation, Formal analysis, Data curation, Conceptualization. **Alexandre Pires Rosa:** Visualization, Validation, Supervision, Project administration, Funding acquisition, Formal analysis, Data curation, Conceptualization. **Cassiane Turchetto Ditadi:** Resources, Methodology, Investigation, Data curation, Conceptualization. **Julia Schneider:** Methodology, Investigation, Formal analysis, Data curation, Conceptualization. **Daniele Cristina de Lima Escrobot:** Writing – review & editing, Project administration, Funding acquisition. **Guilherme Francisco Deda:** Writing – review & editing, Software, Project administration, Formal analysis. **Edita Sostaric Zandee:** Writing – review & editing, Project administration, Funding acquisition.

## Disclosures

The authors declare that they have no known competing financial interests or personal relationships that could have appeared to influence the work reported in this paper.
